# Pathophysiology and Nutritional Approaches in Polycystic Ovary Syndrome (PCOS): A Comprehensive Review

**DOI:** 10.1007/s13668-023-00479-8

**Published:** 2023-05-22

**Authors:** M. Di Lorenzo, N. Cacciapuoti, M. S. Lonardo, G. Nasti, C. Gautiero, A. Belfiore, B. Guida, M. Chiurazzi

**Affiliations:** 1 Department of Clinical Medicine and Surgery, University of Naples “Federico II”, Naples, Italy; 2 Infectious Diseases and Gender Medicine Unit, Cotugno Hospital, AO Dei Colli, Naples, Italy; 3 Department of Medical Oncology, AO “A. Cardarelli”, Naples, Italy

**Keywords:** Polycystic ovary syndrome, Nutritional approaches, Insulin resistance, Hormone profile

## Abstract

**Purpose of Review:**

Polycystic ovary syndrome (PCOS) is the most common endocrine and metabolic disorder in women of reproductive age worldwide. This disease causes menstrual, metabolic, and biochemical abnormalities such as hyperandrogenism, oligo-anovulatory menstrual cycles, polycystic ovary, hyperleptinemia, insulin resistance (IR), and cardiometabolic disorders, often associated with overweight or obesity and visceral adiposity.

**Recent Findings:**

The etiology and pathophysiology of PCOS are not yet fully understood, but insulin seems to play a key role in this disease. PCOS shares an inflammatory state with other chronic diseases such as obesity, type II diabetes, and cardiovascular diseases; however, recent studies have shown that a healthy nutritional approach can improve IR and metabolic and reproductive functions, representing a valid therapeutic strategy to ameliorate PCOS symptomatology.

**Summary:**

This review aimed to summarize and collect evidence about different nutritional approaches such as the Mediterranean diet (MedDiet) and the ketogenic diet (KD), as well as bariatric surgery and nutraceutical supplementation as probiotics, prebiotics, and synbiotics, among the others, used in patients with PCOS.

## Introduction

Polycystic ovary syndrome (PCOS), also known as hyperandrogenic anovulation or Stein–Leventhal syndrome, is a multifactorial and polygenic endocrine disorder, affecting women of reproductive age worldwide [1]. This syndrome is often associated with growth and dysfunctional ovaries, excessive androgen levels, and insulin resistance representing a risk factor for further diseases such as cardiovascular disease and type 2 diabetes mellitus (DMT2) [2, 3], metabolic syndrome (MetS) [3], and depression and anxiety [4, 5].

**Fig. 1:**
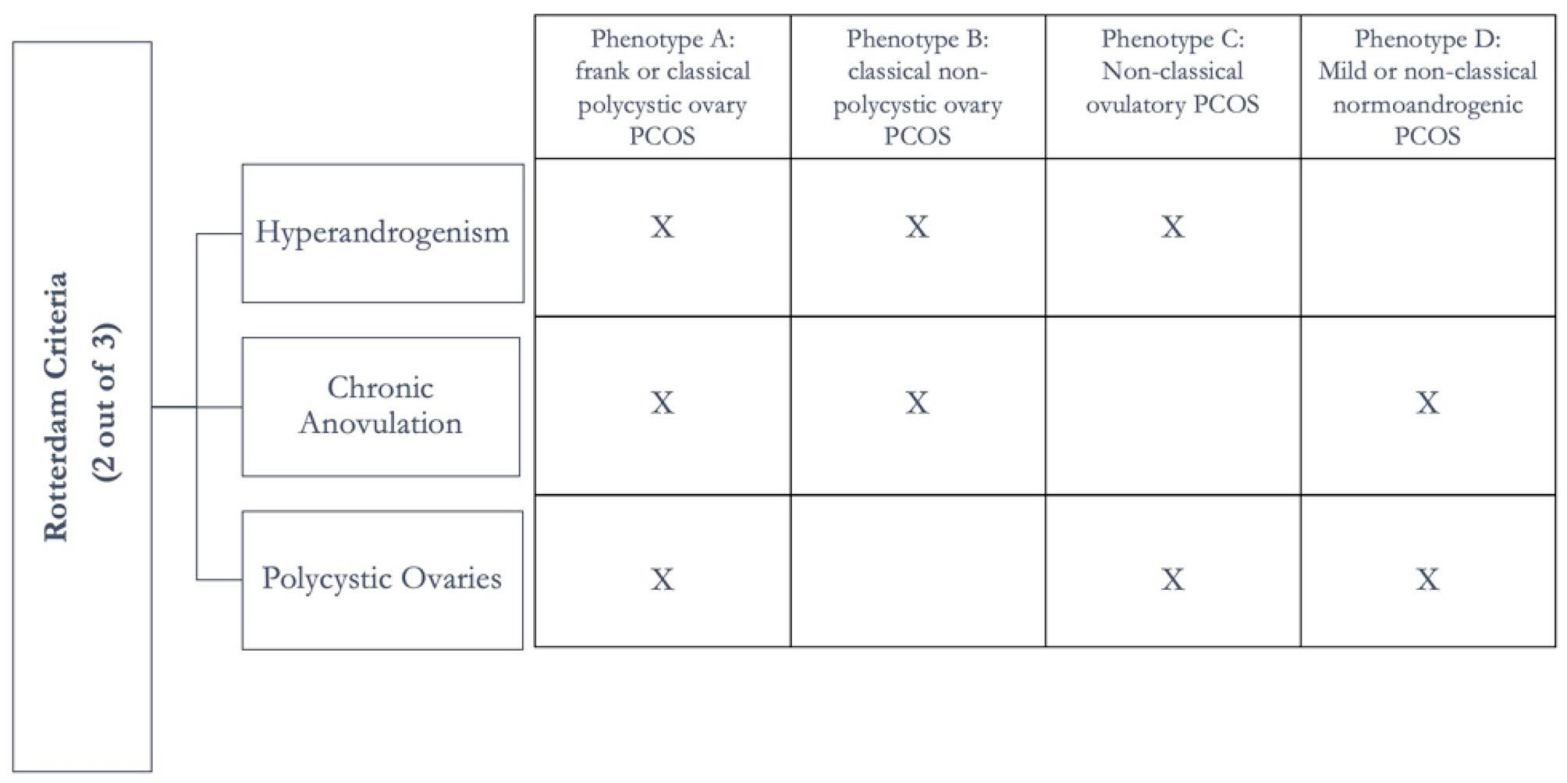
The Rotterdam criteria for the diagnosis of polycystic ovary syndrome

Studies showed 1 out 10 women suffer from PCOS before menopause [6]. In the past, PCOS was considered a disorder of only adult women, but recent evidence showed that PCOS is a lifelong syndrome that can occur from prenatal age. According to the Rotterdam diagnostic criteria (Fig. 1), indeed, the PCOS prevalence in adolescents varies between a minimum of 3% and a maximum of 26% [7]; however, the prevalence of the disease in children is still unknown [8].

Despite a hormonal imbalance related to luteinizing hormone (LH), the underlying causes of PCOS are related also to follicle-stimulating hormone (FSH) and gonadotropin-releasing hormone (GnRH) [9]; the PCOS etiology and pathogenesis are not yet fully understood [2, 9]. Studies suggest a multifactorial etiology involving many different factors such as insulin resistance (IR), hyperandrogenism (HA), and environmental, genetic, and epigenetic factors. Moreover, low-grade chronic inflammation seems to be both cause and effect of the syndrome [10].

## Materials and Methods

A literature search was conducted on the MEDLINE database (accessible through PubMed) for articles in English and published until the year 2023. Medical Subject Headings (MeSH) and keywords terms were used to screen and identify studies. Search Builder with MeSH terms and subheadings contains (((“Polycystic Ovary Syndrome/diagnosis” OR “Polycystic Ovary Syndrome/diet therapy” OR “Polycystic Ovary Syndrome/drug therapy” OR “Polycystic Ovary Syndrome/etiology” OR “Polycystic Ovary Syndrome/metabolism” OR “Polycystic Ovary Syndrome/pathology” OR “Polycystic Ovary Syndrome/physiopathology” OR “Polycystic Ovary Syndrome/prevention and control” OR “Polycystic Ovary Syndrome/surgery” OR “Polycystic Ovary Syndrome/therapy”)) AND “Insulin Resistance”[Mesh]) AND “Diet”[Mesh] (155 items).

MeSH descriptor Polycystic Ovary Syndrome was not exploded all trees (“Polycystic Ovary Syndrome” is the more specific term; we did not explode and click on “Restrict to MeSH terms” and “Do not include MeSH terms found below this term in the MeSH hierarchy”); instead, the other MeSH terms (Insulin Resistance and Diet) were exploded.

Keywords contains “nutritional approaches” OR “insulin resistance” OR “hormone profile.” Non-English articles, published as conference papers or abstracts only, and studies including information that overlapped other publications were excluded. Only articles related to PCOS were included in our search. The selection criteria for the narrative review included original articles (randomized and nonrandomized clinical trials, including prospective observational studies, retrospective cohort studies, and case–control studies) and review articles regarding the influence of different nutritional approaches on PCOS. Articles that met the inclusion criteria were carefully read, and, when appropriate, further articles retrieved from their references were also reviewed with the aim to include other critical studies that might have been missed in the initial search.

## Phenotype

The Rotterdam criteria divided this syndrome into four phenotypes (Fig. 1) [11].

Women with a frank phenotype show a worse profile in metabolic and cardiovascular risk factors than those with a non-classical phenotype despite the same body mass index (BMI) [12]. Similarly, evidence suggests this phenotype may predict a higher postmenopausal cardiovascular morbidity and mortality risk than the non-classical phenotype [13]. Women with the non-classic normoandrogenic phenotype have lower insulin resistance with no PCOS metabolic characteristics than women with the classical or Frank phenotype.

The heterogeneity of PCOS symptoms and manifestations may explain the presence of different diagnostic guidelines; the phenotype can range from being asymptomatic to having all 3 components of the disease (anovulation, hyperandrogenism, and polycystic ovary). So far, different guidelines have led to underdiagnosis or overdiagnosis; for this reason, a new unique guideline, considering all PCOS phenotypes, and the milder forms of this disease are necessary [12, 14, 15].

## Pathophysiology and Diagnosis

Over time, several hypotheses have emerged to explain the pathophysiology of PCOS. In the beginning, an excess of intrauterine androgens was considered able to cause this disease. Consequently, insulin resistance could contribute to PCOS and hyperandrogenemia onset [16]. PCOS is a multifactorial syndrome in which genetic [17–22] and environmental factors contribute to uncontrolled ovarian steroidogenesis, aberrant insulin signaling, and excessive oxidative stress. An intrinsic defect in theca cells could partially explain hyperandrogenemia in PCOS patients; women with PCOS, indeed, present theca cells which, despite the absence of trophic factors, can secrete high levels of androgens due to the intrinsic activation of steroidogenesis [23]. This intrinsic dysregulation can affect granulosa cells which produce up to 4 times higher levels of the anti-Müllerian hormone (AMH) in these patients [24–26]. Studies also show the presence of several follicles, mainly pre-antral and small antral follicles, in females with PCOS [27, 28]. A reduced insulin sensitivity, attributable to a post-receptor binding defect with an alteration in the gene expression of some genes involved in insulin signaling pathways, has also been identified as an intrinsic component of PCOS, regardless of obesity presence [29–31]. Furthermore, PCOS syndrome has been associated to an increase in glycol-oxidative stress [32] secondary to mitochondrial dysfunction, able to induce IR and hyperandrogenism in patients with PCOS [33].

So far, there are no specific diagnostic tests for PCOS diagnosis. Therefore, a careful differential diagnosis plays a relevant role; differential diagnosis related to this investigation seeks to exclude hyperprolactinemia, thyroid disease, Cushing’s syndrome, and adrenal hyperplasia [34, 35]. Pelvic examination, a transvaginal ultrasound, and sex hormone levels measurement associated with a careful medical history, including weight changes and related insulin resistance symptoms, are the most frequently recommended investigations [36]. According to the National Health Service (NHS), irregular or not frequent cycles, high levels of androgens or symptoms related to hyperandrogenism (acne, alopecia, hirsutism), and images showing polycystic ovaries represent specific criteria for the diagnosis of PCOS [37]. Currently, the Rotterdam diagnostic criteria are the most commonly used PCOS diagnosis methods to detect the presence of at least two clinical or biochemical manifestations among hyperandrogenism, ovulatory dysfunction, or polycystic ovaries (Fig. 1) [38].

## Sex Hormone Profile in PCOS

**Fig. 2:**
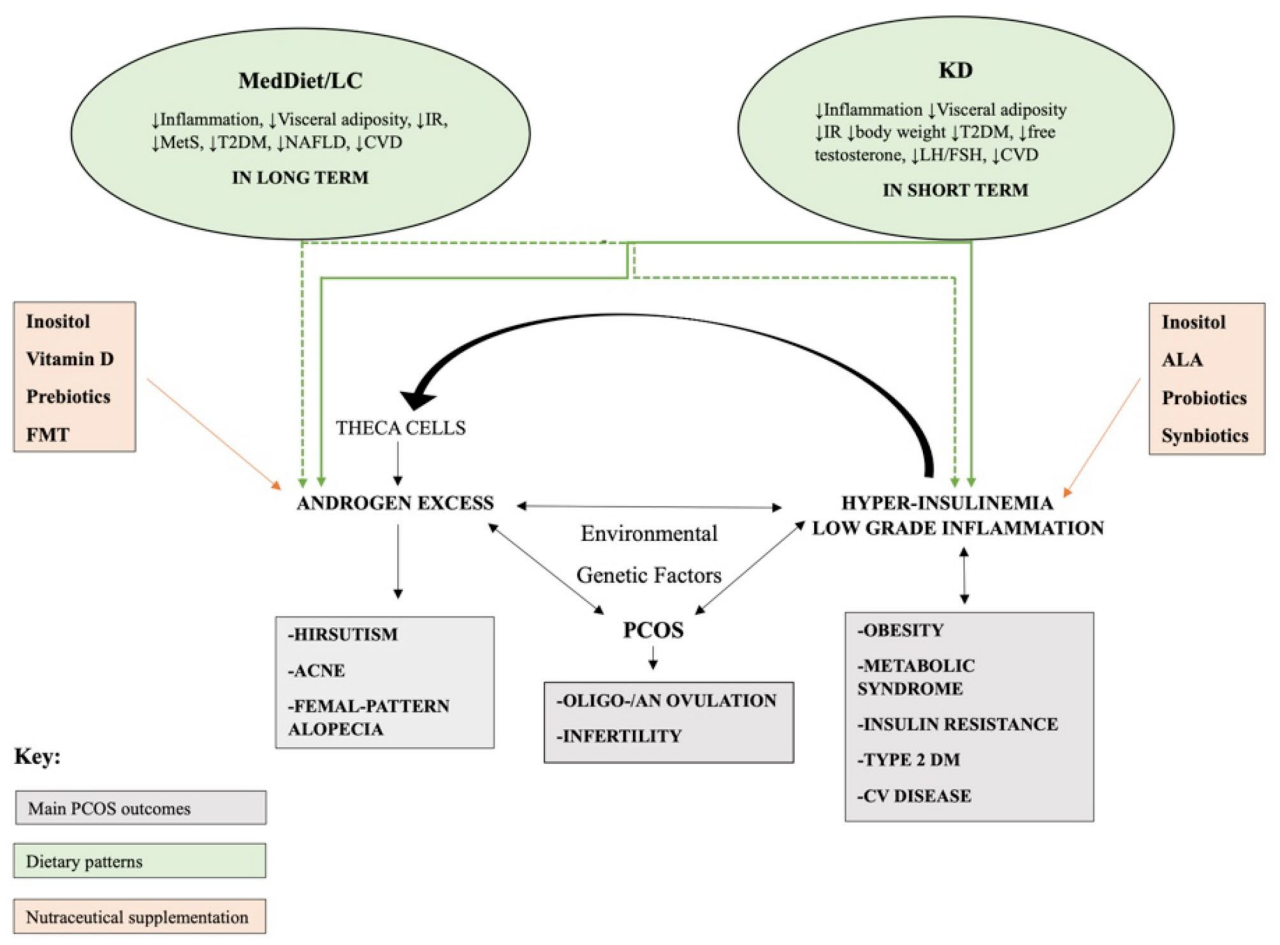
Pathophysiology and potential effects of dietary patterns and nutritional supplements on main health outcomes/risk factors associated with PCOS. MedDiet/LC, Mediterranean diet/low-carbohydrate; KD, ketogenic diet; IR, insulin resistance; MetS, metabolic syndrome; T2DM, type 2 diabetes mellitus; NAFLD, non-alcoholic fatty liver disease; CVD, cardiovascular disease; ALA, alpha-lipoic acid; FMT, fecal microbiota transplantation

A hormonal imbalance, involving the ovaries and the glands that control their activity (hypothalamus and pituitary), is one of the factors involved in the onset of PCOS (Fig. 2). For this reason, during the diagnosis of this disease, the evaluation of different hormones such as LH, as well as FSH, estrone (E1), estradiol (E2), progesterone, testosterone, androstenedione, dehydroepiandrosterone sulfate (DHEA-S), 17-hydroxyprogesterone (17-OHP), sex hormone-binding globulin (SHBG), anti-Müllerian hormone, plays an important role [39].

### LH and FSH

From a neuroendocrine point of view, a distinctive feature of the PCOS syndrome is represented by an inappropriate secretion of gonadotropins, demonstrating the existence of an alteration of the hypothalamus–pituitary–ovary axis characterized by:

- Increase in LH secretion: in particular, there is an increase in the amplitude and frequency of the LH peaks in basal conditions and a hyper-response to the GnRH test. LH gonadotropin can be responsible for the hyperplasia of thecal ovary cells, an anatomopathological substrate that supports hyperandrogenism.
- Normal or reduced levels of FSH: a hypo-functionality of the FSH cells of the ovarian granulosa axis is detected. The FSH low but steady levels continuously stimulate the growth of new follicles; these new follicles cannot reach complete maturation, undergoing atresia and not ovulation. These atretic follicles continue to enrich the ovarian stromal portion that secretes, under the stimulation of LH, a consistent number of androgens.
- Ratio LH/FSH > 2.5: this typical pattern of gonadotropin secretion is the result either of an increased sensitivity of the pituitary gland to hypothalamic GnRH or an altered hypothalamic secretion of GnRH. It has been observed, indeed, that the pulsatile secretion of GnRH can modulate the synthesis of gonadotropins. In particular, a condition of pulsatile secretion of GnRH can stimulate the synthesis of LH, while a low pulsatile secretion would stimulate the synthesis of FSH. In women with PCOS, pulsatile secretion can induce the production of LH. The partial suppression of FSH, the greater sensitivity of this gonadotropin to the negative estrogen feedback, and the relative insensitivity to GnRH appear to be due to the inhibin, whose activity is increased in the ovarian follicles due to the excess of androgens [40, 41].

### Estrogen and Progesterone

In women with PCOS, elevated circulating levels of estrone (E1) and estradiol (E2) corresponding to those of the early follicular phase of eumenorrheic women can be observed, causing an anovulatory condition.

Elevated E1 levels are associated with two biochemical mechanisms:

- Peripheral conversion of androgens (aromatization of androgens in adipose tissue and skin)
- Increased androgen-dependent aromatase activity of granulosa ovary cells

The increased peripheral aromatization of androstenedione to estrone, especially in the adipose tissue, causes an increase in E1 levels and the inversion of the E1:E2 ratio. This state of chronic hyperestrogenism can promote endometrial proliferation and an increased risk of endometrial cancer [42].

Additionally, the syndrome is characterized by decreased progesterone secretion due to chronic anovulation. Furthermore, exposure to high estrogen levels unbalanced by appropriate progesterone levels may predispose to the development of atypical endometrial hyperplasia [43].

### Hyperandrogenism

Biochemical and clinical hyperandrogenism of ovarian and adrenal origin is observed in about 60–80% of patients with PCOS, thus resulting in one of the main characteristics of the syndrome, even if its presence is not essential for PCOS to diagnose according to the Rotterdam criteria (Fig. 1) [44].

Hyperandrogenism of ovarian origin is mainly due to defective intrinsic steroidogenesis in theca cells [25, 42] resulting from increased activity of enzymes that catalyze several steps of androgen synthesis such as:

- Cholesterol-desmolase (CYP11A1): responsible for converting cholesterol to pregnenolone
- 17α-Hydroxylase and 17,20-lyase: able to convert pregnenolone to 17-OH-pregnenolone and then to dehydroepiandrosterone (DHEA)
- 3-β-Hydroxydodehydrogenase: able to convert pregnenolone to progesterone, 17OH-pregnenolone to 17OH-progesterone, and DHEA to androstenedione

The increased function of these steroidogenic enzymes is determined by both extra- and intra-ovarian factors; in particular, regarding extra-ovarian factors, the increased pulsatile secretion of LH leads to constantly increased levels of circulating LH that stimulates the thecal synthesis of androgens. These high levels of LH are also partly due to altered negative feedback from androgens on the hypothalamus–pituitary axis. The relatively low levels of FSH related to LH, play an indirect role in stimulating aromatase to a lesser than normal extent, resulting in reduced conversion of androgens to estrogens, exacerbating hyperandrogenism [45]. Insulin, acting on its receptors at the ovarian theca level, also represents a trigger capable of leading in synergy with LH to an increase in thecal steroidogenesis by stimulating the expression of CYP17α1 mRNA and its enzymatic activity.

Also, the hormones generated by the granulosa cells, such as the AMH and inhibin, contribute to the altered steroid-genetic activity of the theca cells. AMH exerts a direct paracrine effect by stimulating androgen production and indirectly inhibiting the action of FSH on aromatase-by-aromatase inhibition [45, 46].

Abnormalities in adrenal steroidogenesis contribute minimally to adrenal hyperandrogenism and are also due to cytochrome P450-17alpha-hydroxylase (CYP17α1) hyperactivation [47]. An additional role would seem to be played by an increased peripheral metabolism of cortisol: the reduced cortisol levels would cause inadequate negative feedback on the hypothalamus–pituitary–adrenal axis with greater production of adrenocorticotropic hormone (ACTH) at the pituitary level and stimulation of the adrenal gland at the steroidogenesis [48, 49].

Hyperandrogenism must be assessed by measuring the levels of:

- Total testosterone
- Androstenedione
- Dehydroepiandrosterone sulfate
- Free androgens → calculated determination of free testosterone levels or through the free androgen index (FAI)

The determination of free testosterone can be inaccurate; for this reason, the Consensus Conference in Rotterdam established that the calculation of the FAI should be preferred since it has greater sensitivity and specificity [50].

### SHBG and AMH

SHBG is a protein produced in the liver and able to bind sex hormones (androgens and estrogens), regulating their bioavailability to target tissues. In patients with PCOS, there is an approximately 50% reduction in SHBG compared to its normal levels and a consequent increase in free androgens.

The levels of AMH in women with PCOS are increased compared to women without PCOS, and this increase would be proportional to the clinical severity of the syndrome. Scientific evidence suggests that this increase is due to the stimulus exerted by androgens on the early stages of follicular growth. It has been shown that the increase in serum levels of AMH progress as the same of the androgens, for which AMH has been proposed as a marker of hyperandrogenism of ovarian origin [51].

## PCOS and Insulin Resistance

It has been estimated that about 75% of subjects with PCOS have insulin resistance (IR) [52].

Insulin can regulate glucose homeostasis by suppressing hepatic glucose production [53] or stimulating glucose uptake by insulin-responsive target tissues such as adipocytes and cardiac and skeletal muscle. Furthermore, insulin suppresses lipolysis, resulting in a decrease in the level of circulating free fatty acids that can mediate the action of insulin on the hepatic production of glucose [54]; however, insulin exerts some other metabolic, mitogenic, and reproductive functions [55].

IR is characterized by increased insulin circulating levels both basally and after glycemic load; it consists of an inability of insulin to mediate the actions related to the production and uptake of glucose and/or lipolysis with a consequent request of a greater amount of insulin to obtain a certain metabolic action [56]. This condition plays a key role in the development of PCOS and can induce several metabolic and reproductive abnormalities in women with this syndrome [57, 58].

Furthermore, IR and associated hyperinsulinemia are related to abnormal ovarian steroidogenesis [59] and can concur to the pathogenesis of anovulation and hyperandrogenism [60, 61]; in particular, hyperinsulinemia stimulates theca cell proliferation, amplifies LH-mediated androgen secretion, and increases expression of LH and insulin growth factor-1 (IGF-1) receptor. Wallace et al. in 2013 [62] observed that high insulin levels inhibit the production of SHBG by the liver, causing increased levels of free testosterone; moreover, IGF-BP1 synthesis was inhibited, increasing the level of free IGF-1 [63].

To date, right temporal relationship between PCOS and insulin resistance is still unknown; several studies have tried to identify the possible mechanisms involved in the IR-PCOS relationship suggesting an involvement of the insulin transduction pathway (Fig. 2).

Reduced binding of insulin to pancreatic β-cells could be due to a reduced abundance of GLUT4 glucose transporter that in turn leads to low glucose uptake and a decreased sensitivity to insulin can explain IR [64–67].

Further evidence has shown a post-binding defect in the early steps of insulin signal transduction especially in adipocytes [68, 69] and skeletal muscle [59, 70], probably due to a marked increase in insulin-independent receptor phosphorylation [59], such as insulin receptor substrates 1 (IRS-1) and phosphorylation or activation of phosphatidylinositol 3-kinase (PI3-K) [71]. Moreover, in skeletal muscle, kinases involved in the MAPK-ERK1/2 mitogenic pathway are constitutively activated, contributing to IRS-1 phosphorylation and metabolic signaling inhibition [72, 73].

Another possible hereditary cause of IR in PCOS females is a significant rate of SH2 domain-containing adaptor protein (Lnk) activity in ovarian cells that suppresses the MAPK-ERK and phosphatidylinositol 3-kinase-AKT signaling responses to insulin [74].

On the other hand, it has been suggested that molecular defects underlying IR in PCOS may not be related to alterations in insulin signaling pathways [75] but to an alteration in plasma levels of adiponectin [76], which plays a modulating role in human skeletal muscle through AMPK [77].

Excessive body fat, another common feature in PCOS women, can also contribute to worsening the entire clinical picture associated with IR, despite IR can be found also in normal-weight women [78]. It has been observed, indeed, that a high BMI, with an increase in fat mass, mainly in the abdominal area, is responsible for the IR’s worst effects [79, 80], and this could be due to the presence of dysfunctional adipocytes [81] and to the production of adipokines by the subcutaneous and visceral fat [82].

Nowadays, the association between obesity and inflammation is validated, and low-grade chronic inflammation may contribute to IR by the activity of inflammatory adipocytokines, such as TNF-α [83, 84] whose circulating levels are increased in PCOS patients [85]

Moreover, since obesity and insulin resistance are often associated with type 2 diabetes (T2D), the likelihood of observing PCOS in a patient with diabetes is very high and often associated to pancreatic β-cell dysfunction [86–89].

Furthermore, in 2013, Dalamaga M et al., in a prospective controlled study of patients diagnosed with PCOS, demonstrated that ovarian SAHA syndrome (seborrhea, acne, hirsutism, and androgenetic alopecia) is associated with a higher IR profile representing a risk factor independent for glucose abnormalities. Furthermore, it would appear that patients with PCOS and SAHA syndrome more frequently exhibit the severe PCOS phenotype associated with the triad of OA (anovulation and or/oligoanovulation), hyperandrogenemia, and PCO. Their results support an independent association between ovarian SAHA syndrome and risk of glucose abnormalities suggesting that prompt recognition of SAHA syndrome in women with PCOS allows for earlier diagnosis of metabolic abnormalities and closer surveillance of women whose metabolic profile indicates potential risks of adverse health outcomes [90].

## Dietary Models and PCOS

The management approach and the choice of the best therapeutic option depend on the patient and her priorities [9]. Complications can vary from seeking fertility to regulating menstrual disorders, weight loss, or relief of hyperandrogenic symptoms [90]. Therefore, the approach should be individual to achieve the best result for each patient. Up to date, there is still no ideal or definitive treatment for this condition; for this reason, the current approach is characterized by a symptomatic therapy with many drugs, including oral contraceptives, insulin sensitizers, cyclic progestins or antiandrogens, and fertility treatments associated with lifestyle changes [91, 92••].

Environmental factors such as eating habits play an important role in the PCOS prevention and treatment; lifestyle changes, healthy nutrition, and adequate body weight achieving or maintaining are the most important therapeutic strategies in these patients. Specifically, the dietary approach in these women must be aimed to achieve specific goals such as improving IR and metabolic and reproductive functions [93].

Several studies have identified different dietary approaches among women with PCOS and without PCOS; in particular, women with PCOS showed an increased intake of calories and saturated fat and inadequate consumption of fiber, suggesting that the clinical symptoms and the combined risk of chronic diseases in these patients could be exacerbated by the unhealthy diet. In 2017, Szczuko et al. [94] analyzed the diet of 54 childbearing-age women with PCOS, showing that poor diets may be the cause of metabolic disorders related to improper function of the ovaries in women with PCOS.

In 2009 Chavarro et al. [95], studying a cohort of apparently healthy pre-menopausal women concerning the risk of ovulatory infertility, found that greater carbohydrate intake and dietary glycemic load were associated with an increased risk of infertility due to anovulation and that dietary glycemic index was positively associated to infertility among nulliparous women in this cohort (Table 1).

An increased prevalence of eating disorders has been observed in women with PCOS, suggesting that all women with this syndrome should undergo routine screening at the first diagnosis [96]. To date, international guidelines for the PCOS assessment and management declare that all women with PCOS should follow a healthy lifestyle for life; on the contrary, dietary approaches aimed to induce weight loss should be recommended to overweight or obese women with PCOS [97] (Fig. 2).

### Mediterranean Diet

To date, the Mediterranean diet (MedDiet) is the gold standard dietary model in preventive medicine due to its anti-inflammatory, antineoplastic, antiobesogenic, and antioxidant properties [98]; thus, it has been included in the international guidelines among the recommended dietary models due to its unique characteristics, including the regular consumption of unsaturated fats, fibers, low-carbohydrate glycemic index, antioxidants, and vitamins, as well as adequate amounts of animal and vegetable proteins [98]. MedDiet gained international recognition through the work of Ancel Keys and is considered the original prototype for current dietary guidelines in the USA and other countries. One of the first definitions of the Mediterranean diet was provided by Willet et al. who asserted that the Mediterranean diet reflects the typical eating patterns of some regions of Greece and Italy in the early 1960s, where the adult’s life expectancy was remarkably high, while the rates of diet-related chronic disease were low [99••]. Several studies, indeed, over the years, have shown that the adoption of the MedDiet can protect against diseases, such as obesity, cardiovascular disease, type 2 diabetes (T2D), and non-alcoholic fatty liver disease (NAFLD) [100, 101].

The beneficial mechanisms of MedDiet involve the reduction of inflammatory and oxidative stress markers and the improvement of lipid profiles, insulin sensitivity, and endothelial function, as well as antiatherosclerotic and antithrombotic properties [100, 101]. Furthermore, MedDiet is also considered the best dietary model for the primary prevention of MetS [101]. Considering the close relationship between PCOS and obesity, low-grade chronic inflammation, and IR, MedDiet represents one of the optimal non-drug strategies for the PCOS treatment. MedDiet is typically based on plant-based foods, including vegetables, fruits, whole grains, nuts, and seeds. These provide antioxidants, and significant amounts of fiber, as well as vitamins and minerals. Healthy lipids are another significant dietary benefit of MedDiet, particularly those derived from olives, nuts, and fish such as salmon and sardines. These sources are rich in heart-healthy monounsaturated fats and are often used to replace the saturated and trans fats of fatty meats and cheeses. Moderate amounts of dairy products, fish, and poultry and lower levels of red meat are consumed. Additionally, spices and herbs are commonly used to flavor foods to avoid overdoing the salt. The beneficial effects of MedDiet have been attributed to plant polyphenols. Vegetable polyphenols obtained from vegetables, fruits, legumes, cereals, nuts, seeds, and especially red wine and extra virgin olive oil in the MedDiet may be used to counteract MetS and have been scientifically studied in the last decades [102]. Polyphenols seem to have a possible role in disease prevention and have therapeutic potential in women with PCOS, slowing the progression of inflammation and improving both insulin sensitivity and compensatory hyperinsulinemia [103]. In conclusion, the beneficial effects of MedDiet can be attributed to various foods that exhibit anti-inflammatory and antioxidant properties [104]. In 2019, Barrea et al. [99••] evaluated MedDiet adherence, dietary intake, body composition and their association with the clinical severity of PCOS in a cohort of 112 PCOS-naïve women compared to a control group of healthy women matched by age and BMI. Although there was no difference in energy intake between the two groups, women with PCOS consumed fewer complex carbohydrates, fiber, monounsaturated fatty acids (MUFA), and n-3 polyunsaturated fatty acids (PUFAs) and more simple carbohydrates, saturated fatty acids (SFAs), and n-6 PUFAs compared to the control group, suggesting a new direct association between MedDiet adherence and clinical disease severity in women with PCOS. Furthermore, women with PCOS showed a different body composition than controls, with lower phase angle (PhA) and lean mass values. These data could support the therapeutic role of the single foods and nutrients of the Mediterranean dietary pattern in women with PCOS, helping to reduce the inflammatory state linked to IR and hyperandrogenemia. They also suggest that PhA could represent a useful marker of the clinical severity of PCOS, prompting that nutritional and body composition assessment in women with PCOS can be an important strategy in the management of this syndrome. In 2022, Mei et al. in a 12-week randomized controlled clinical trial evaluated the therapeutic effect of a Mediterranean diet combined with a low carbohydrate (MedDiet/LC) dietary pattern or low-fat (LF) diet in 72 overweight patients with PCOS. Their results showed that patients belonging to the MED/LC group and treated with a maximum carbohydrate intake of less than 20%, a maximum carbohydrate intake of 100 g throughout the day, and an increased intake of protein and fat showed an improvement in restoring the menstrual cycle as well as in anthropometric parameters, reproductive endocrine levels, IR levels, and plasma lipid levels compared to patients belonging to LF group and treated with less than 30% of total dietary calories from fat, less than 40 g of fat intake throughout the day, and up to 10% saturated fat. These data suggest that the MED/LC diet model can be used in the clinical treatment of patients with overweight PCOS [105] (Table 1).

### Ketogenic Diet

The ketogenic diet (KD) is an isocaloric diet, high in fat, low in carbohydrates (CHO), and normoproteic. The therapeutic role of KD has been studied for a long time, and several papers have supported the thesis that physiological ketosis may be useful in many pathological conditions, such as epilepsy, neurological diseases, cancer (with a ketogenic isocaloric diet) or obesity, type 2 diabetes, acne, and respiratory and cardiovascular diseases (with a generally low-calorie ketogenic diet) [106••]. Nutritional ketosis represents the ultimate goal of ketogenic diets and is characterized by a diet rich in fat, adequate protein, and very-low carbohydrates that mimics the metabolism of the fasted state to induce the production of ketone bodies [107].

There are no more evidence showing the effects of KD on PCOS. In 2005, Mavropoulos et al. [108], in a small uncontrolled pilot study, showed a significant reduction in body weight, free testosterone, LH/FSH ratio, and fasting insulin after a KD regimen, suggesting favorable effects on both anthropometric and metabolic features in affected patients.

In 2020, Paoli et al. [106••] studied 14 overweight women diagnosed with PCOS who observed a modified KD (KEMEPHY diet, a Mediterranean eucaloric ketogenic protocol (about 1600/1700 kcal /day) with the use of some plant extracts. Twelve weeks after the dietary intervention, these patients showed a significant reduction in body weight and BMI, as well as a reduction in fat mass, visceral adipose tissue, and a marked improvement in IR. In addition, an improvement in the hormonal picture (LH, LH/FSH ratio, testosterone, SHBG, E2, progesterone) was observed in these women. Similar results were obtained from Cincione et al. in 2021, suggesting that KD may represent an optimal dietary intervention for patients with PCOS [109].

Considering the potential side effects of a high-fat diet, a very-low-calorie KD (VLCKD), characterized by a low-fat count, mainly derived from olive oil, as in the MedDiet, could represent an alternative strategy to KD, and it could help these patients lose weight and improve symptoms.

In 2022, Magagnini et al. studied the effect of a 3-months VLCKD, in a group of 25 obese women with PCOS, on ovarian reserve and luteal function in women with PCOS, founding that there was a metabolic and ovulatory improvement, achieved in a relatively short time [110].

Although short-term KD seems to be effective, PCOS is a chronic disease requiring long-term treatment, and animal experiments suggest that long-term maintenance of KD can affect the metabolic status and stimulate the development of NAFLD and systemic glucose intolerance [111] (Table 1).

### Bariatric Surgery

Bariatric surgery appears to be the main option for weight loss, in a short time, in severely obese individuals; recently, it has been used in obese women with PCOS. In particular, bariatric surgery promotes significant weight loss, which is associated with improvement in IR, hyperandrogenism, menstrual irregularity, and ovulatory dysfunction. Therefore, surgery can successfully mediate the regression of PCOS and promote fertility [112]. In 2021, Ezzat et al. evaluated the effects of weight reduction achieved by bariatric surgery on androgen levels and ovarian volume by ultrasound in 36 obese patients with polycystic ovaries. The results obtained showed a significant reduction in body mass index, free and total serum testosterone levels, an increase in SHBG and menstrual cycle regulation at 6 and 12 months after the operation, and a reduction in both free androgen index and ovarian volume on ultrasound [113]. Therefore, bariatric surgery should be considered as a possible treatment in obese patients with PCOS, especially in those with MetS [112]. Likewise, in 2022, Hu et al. evaluated the difference in efficacy between drug and bariatric surgery therapy in 90 women with obesity and PCOS suggesting that bariatric surgery should be considered as the first-line treatment for patients with PCOS and obesity, being far more effective than drug therapy [114••]. However, further, more comprehensive and longer follow-up studies are needed to investigate the role of bariatric surgery in obese women with PCOS (Table 1).

### Nutraceutical Supplementation

Women with PCOS often show a deficiency of many common nutrients, vitamins, and minerals associated with the psychological sequelae of the condition such as depression or anxiety, as well as physiological sequelae such as insulin resistance, diabetes, and infertility [115]. In recent years, several evidences have reported that nutraceutical supplementation represents a promising and safe therapeutic strategy for PCOS women. Thus, nutrient supplementation in addition to traditional lifestyle-based therapy in PCOS may benefit these women. Several studies have highlighted that inositol, a carboxylic sugar belonging to the complex family of vitamin B, is a useful molecule able to counteract the clinical and metabolic signs of PCOS [116]. Specifically, two stereoisomers of inositol, the myo-inositol, and D-chiro-inositol (DCI), seem to play a main role in exerting several pleiotropic actions, including insulin-dependent androgen synthesis [117], modulation of insulin transduction, and glucose metabolism [116].

Another molecule of interest is alpha-lipoic acid (ALA), largely present in potatoes, broccoli, spinach, tomatoes, Brussel sprouts, peas, brown rice, and red meat. Humans absorb only a few ALA amounts in biologically active form; it is, indeed, rapidly metabolized and therefore does not accumulate in human tissues [118]. ALA is a potent-free radical scavenger and exerts insulin-sensitizing activity and could be useful in the PCOS treatment [119] even if its beneficial effects are only regarding metabolic features of the syndrome [120]. Studies have suggested that ALA can reduce body weight by affecting food intake and increasing energy expenditure by suppressing hypothalamic AMPK activity. In 2010, Masharan et al. demonstrated that 600 mg twice daily of controlled-release alpha-lipoic acid (CRLA) administered for 16 weeks in 6 women with PCOS could induce an improvement in IR and plasma lipid profile, suggesting that the CRLA has positive effects on the PCOS phenotype [121].

The worth of interest is the promising therapeutic strategy that involves the combination of inositol plus ALA, exerting a synergistic action in improving glycemic control, IR, and metabolic and endocrine features in PCOS patients [122–124]. In 2015, Cianci et al. examined the role of the combination of DCI and ALA in 46 women with PCOS suggesting that the association might have a strong impact on a metabolic profile even with a short-term treatment. Further studies on alpha-lipoic acid are needed to clarify the impact of ALA in PCOS [124].

Some studies show a beneficial effect of vitamin D on the improvement of glycemic metabolism in women with PCOS who showed a vitamin D deficiency; on the contrary, further studies are needed to evaluate a possible beneficial activity on the plasma lipid profile, inflammation, and hyperandrogenism of women affected by PCOS [125–127]. According to papers in the literature, supplementation with complementary nutrients and therapies can be able to improve some of the adverse health outcomes associated with PCOS. However, more research is needed to determine the efficacy of these therapies and their actions and interactions with the biological processes underlying PCOS (Table 1).

### Probiotics, Prebiotics, and Synbiotics

Some evidence has led to postulate the hypothesis that alterations in the microbiome are involved in the genesis of PCOS. Indeed, it has been observed that the gut microbiome of women with PCOS appears to be less diverse and with greater intestinal permeability than in women without PCOS; these characteristics are closely related to hyperandrogenism and increased levels of systemic inflammation [128, 129].

Treatment options for the altered gut microbiome causing PCOS include probiotics, prebiotics, synbiotics, and more recent therapies, including fecal microbiota transplantation (FMT) [130].

Probiotics naturally occur in fermented foods and are “live microorganisms that, when administered in appropriate amounts, confer a health benefit to the host” [131]. In women with PCOS, therapy with probiotics results in an improved metabolic profile. In fact, it has been seen that supplementation with *L. casei*, *L. acidophilus*, and *B. bifidum* for 12 weeks is capable of leading to a reduction in BMI with favorable effects on glycemia and very-low-density lipoprotein (VLDL), and triglycerides in PCOS patients [132]. Similarly, a significant reduction in plasma glucose and serum insulin levels was observed in PCOS women treated for 8 weeks with supplementation of *L. casei*, *L. acidophilus*, *L. rhamnosus*, *L. bulgaricus*, *B. breve*, *B. longum*, and *S. thermophiles* [133]. Shamasbi et al. in a recent meta-analysis have shown that probiotics have a significant impact on the hormonal profile of women with PCOS with a significant decrease in androgen index (FAI) and malondialdehyde (MDA) and an increase in SHBG and nitric oxide (NO) [133].

Prebiotics are fermented substances that cause specific changes in the composition and/or activity of a host’s gut microbiota; the most known ones are inulin, lactulose, fructooligosaccharides (FOS), and galactooligosaccharides (GOS) [131]. Since prebiotics induce the growth of both *Bifidobacterium* and *Lactobacillus*, they produce positive effects on immunomodulatory properties and metabolic markers by producing an important reduction in the levels of glucose, triglycerides, total cholesterol, and LDL [134]. In 2018, Shamasbi et al. have seen that regular consumption of resistant dextrin, which is a prebiotic, may help to regulate metabolic parameters and reduce hyperandrogenism, hirsutism, and menstrual cycle abnormalities in PCOS women [135].

Synbiotics refer to dietary supplements composed of probiotics and prebiotics: compounds in food that stimulate the growth and activity of probiotics [136]. In 2020, Cozzolino M. et al., in a meta-analysis, showed that probiotic/symbiotic administration can improve metabolic, hormonal, and systemic inflammatory factors in women with PCOS. Probiotics and synbiotics, indeed, seem to significantly reduce fasting plasma glucose, fasting blood insulin, HOMA IR, and triglycerides. Furthermore, probiotics and synbiotics also seem to have an impact on anthropometric parameters such as BMI and body weight in women with PCOS, through a positive modulation of energy balance, supported by a reduction in circulating leptin levels after treatment [137]. According to papers in the literature, supplementation with probiotics, prebiotics and synbiotics can be able to improve some of the adverse health outcomes associated with PCOS. However, more research is needed to determine the duration and doses of these therapies and to evaluate their actions and interactions with the biological processes underlying PCOS (Table 1).

**Table 1:** Main prospective studies and meta-analyses included in the review

**First author, year of publication**	**Study type & design**	**Population**	**Results & major findings**
**Lei et al. 2020**	Systematic review and meta-analysis (just published and ongoing randomized controlled trials (RCTs) and human studies are included)	Adult women diagnosed as PCOS Ethnicity: regardless	Inositol and ALA combination is likely to act as a promising and safe therapy for PCOS women IR and oxidative stress status improvement
**Barrea et al. 2019**	Cross-sectional, observational study	112 patients with PCOS, aged 18 to 40 years old (yo) Ethnicity: Caucasian	Association between the adherence to MD and the clinical severity of PCOS A role of PhA as a useful marker of the clinical severity of PCOS
**Mei et al. 2022**	Open-label, parallel-group randomized controlled trial	72 patients with PCOS (aged 16 to 45 yo): 36 patients to low-fat (LF) diet group, 36 patients to MD/low carb (MD/LC) diet group Ethnicity: Asiatic (China)	Effectiveness of the LF and MED/LC dietary models in modifying anthropometric parameters, reproductive endocrine levels, IR levels, and lipid levels The MED/LC diet model was recommended for the treatment of overweight patients with PCOS
**Paoli et al. 2020**	12 weeks, single-arm study (interventional)	24 overweight women with PCOS, aged 18 to 45 yo, followed a Ketogenic diet (KD) for 12 weeks Ethnicity: not declared	KD as a possible therapeutic aid in PCOS
**Mavropoulos et al. 2005**	Pilot study (interventional)	11 women with PCOS, aged 18 to 45 yo, BMI ≥ 27 kg/m^2^ Ethnicity: not declared	A LCKD led to significant reductions in weight, percent free testosterone, LH/FSH ratio, and fasting serum insulin in women with obesity and PCOS over a 6-month period
**Cincione 2021**	Interventional study	17 overweight and obese women with PCOS, aged 18 to 45 yo, treated for 45 days with modified KD protocol, defined as “mixed ketogenic” Ethnicity: not declared	KD improves the anthropometric and many biochemical parameters (LH, FSH, SHBG, insulin sensitivity and HOMA index) and reduces androgenic production Improvement of the LH/FSH ratio
**Magagnini et al. 2022**	Retrospective study	25 women, aged ≥ 18 yo, with PCOS and first-degree obesity, who underwent VLCKD-protocol (3 phases of VLCKD, 3 phases of low-calorie diet and 2 phases of maintenance diet that consisted of a balanced diet; each phase lasted 4 weeks) Ethnicity: Caucasian	Metabolic and ovulatory improvement is achieved in a relatively short time
**Ezzat et al. 2021**	Cohort study	36 infertile women with PCOS, aged 22 to 40 yo, who underwent bariatric surgery Ethnicity: not declared	At 6 and 12 months post-bariatric surgery ↓ BMI, free, and total serum testosterone levels and free androgen index ↑ SHBG, menstrual cycle regularity
**Lili Hu et al. 2022**	Single-center, prospective, nonrandomized trial	90 women with PCOS, aged 18 to 40 yo, BMI ≥ 27.5 kg/m^2^, divided in two groups: treated with drugs or bariatric surgery 12-month follow-up Ethnicity: not declared	Bariatric surgery should be the first-line treatment for patients with PCOS and obesity
**Masharani et al. 2010**	Interventional study	6 non-obese women with PCOS, aged 23 to 24, were administered controlled-release ALA (CRLA) 600 mg twice daily for 16 weeks Ethnicity: not declared	↑ insulin sensitivity ↓ triglyceride plasmatic levels ↓ LDL
**Genazzani et al. 2017**	Interventional study	32 overweight/obese PCOS women, aged 23 to 26 yo, were administered alpha-lipoic acid (ALA) (400 mg) once a day for at least 3 months Ethnicity: not declared	↓ insulin, glucose, BMI, and HOMA index, hyperinsulinemia, and insulin response to OGTT. The result of this study sustains the major role of ALA treatment on PCOS metabolic disease
**Cianci et al. 2015**	Prospective study, randomized controlled trial	46 women with PCOS, aged 16 to 32 yo, were divided in two groups: study group (n. 26, 1000 mg D-chiro-inositol and 600 mg ALA daily) and control group (n. 20, untreated). Treatment was taken for 180 days Ethnicity: Caucasian	Clinical and metabolic aspects of women of study group improved compared to the control group. No statistically difference was observed in total cholesterol and triglycerides levels in both groups at follow-up
**Miao et al. 2020**	Meta-analysis	11 studies involving 483 participants Ethnicity: regardless	No positive effect of vitamin D supplementation on BMI, dehydroepiandrosterone sulfate, triglyceride levels, or high-density lipoprotein-cholesterol Vitamin D supplementation reduced insulin resistance and hyperandrogenism, as well improved the lipid metabolism of patients with PCOS. Therefore, vitamin D should be considered as a treatment for PCOS
**Lindheim et al. 2017**	Pilot cohort study	16S rRNA gene amplicon sequencing was performed on stool samples from 24 PCOS patients and 19 healthy controls, all aged ≥ 18 yo and pre-menopausal Ethnicity: not declared	PCOS patients have a lower diversity and an altered phylogenetic profile in their stool microbiome, which is associated with clinical parameters, compared to healthy group
**Torres et al. 2017**	Cohort study	163 pre-menopausal women all aged ≥ 18 yo: healthy women (n. 48), women with polycystic ovarian morphology PCOM (n. 42), and women diagnosed with PCOS using the Rotterdam criteria (n. 73) Ethnicity: not declared	Lower α diversity in PCOS women vs healthy women. PCOM women showed an intermediate (between that of the other two groups) change in α diversity. Hyperandrogenism, total testosterone and hirsutism were negatively correlated with α diversity. Hyperandrogenism was also correlated with β diversity
**Cozzolino et al. 2020**	Systematic review and meta-analysis	9 RCTs involving 587 PCOS women treated with probiotics or synbiotics (at least 8 weeks) or without therapy were included Ethnicity: regardless	In treated women: Improvement of metabolism, reduction of serum testosterone and systemic inflammation

An emerging therapeutic strategy for PCOS is represented by FMT that consists of the infusion of microorganisms from the feces of healthy donors in order to obtain a rapid change in the composition of the gut microbiome of the host [138] (Table 2).

**Table 2:** Summary of therapeutic effects of supplementation with probiotics, prebiotics, and symbiotic

**Therapy**	**Study model**	**Effects**	**References**
Probiotic	Human	↓BMI ↓glycaemia, ↓insulin, ↓ triglycerides, ↑ HDL Positive control of hormonal and inflammatory indicators	[132, 133]
Prebiotic	Human	↓glycaemia, ↓insulin, ↓ triglycerides, ↓ LDL ↓hyperandrogenism, ↓hirsutism, ↓menstrual cycle abnormalities Positive effects on immunomodulatory properties and metabolic markers	[134, 135]
Synbiotic	Human	↓BMI and body weight ↓glycaemia, ↓insulin, ↓HOMA-IR, ↓triglycerides Positive control on hormonal, metabolic, and inflammatory markers	[136, 137]

To date, there is available data regarding the use of FMT to treat PCOS only in murine models where it has been seen that this treatment is associated with a decrease in serum androgen levels and an increase in estrogen levels and normalization of the ovarian cycle [139]. In the light of the above data, prospective results from laboratory studies should encourage further investigations on humans.

## Shortcomings, Challenges, and Future Perspectives

One of the main challenges in the diagnostic–therapeutic course of PCOS is the identification of phenotype and the main cause of the disorder. This is a multifactorial etiology condition, often accompanied by signs and symptoms that cannot be directly ascribed to a metabolic alteration.

For example, it is important to screen women with PCOS for all complications, including dyslipidemia and psychological distress [140].

Generally, treatment is focused on alleviating symptoms, which can differ substantially among PCOS phenotypes; the woman’s needs such as symptoms that cause her the most discomfort or the pregnancy seeking, among the others, cannot be disregarded. Women with PCOS often report significant dissatisfaction with the diagnostic process, the information provided, and the conventional treatment prescribed; moreover, several studies even found increased psychological distress after diagnosis [141].

An additional challenge is choosing the most appropriate nutritional strategy for weight loss, as well as maintaining weight loss.

Women with PCOS, indeed, have additional difficulties in weight loss and maintenance, including insulin resistance, androgen excess, and impaired appetite regulation.

Furthermore, there are normal-weight women with PCOS who, however, have been shown to have greater visceral adiposity than normal-weight controls without PCOS [142]; hence, body re-composition and weight gain prevention interventions should be carried out [143].

It is important, therefore, to dialog with patients and make them understand that PCOS is a long-term illness where it would be desirable to seek a balance between treatment and daily life, in particular regarding nutritional management [141].

The study of new pharmacological and nonpharmacological therapeutic strategies to provide better treatment of PCOS is the aim of the research, but, to date, studies would seem to be limited by methodological problems, different diagnostic criteria, small sample size, nonrandomized design, and short follow-up [143].

Moreover, not all outcomes have been adequately studied. The weight loss interventions’ effectiveness on reproductive function, fertility outcomes, cardiovascular and psychological health, quality of life, and appetite regulation still requires larger and sufficiently powered studies [143].

However, a meta-analysis of lifestyle interventions [144] showed an improvement in the free androgen index by reducing body weight (low-quality evidence), but no specific impact on childbirth or menstrual regularity was detected [140].

In addition to standard drug therapies, including the estroprogestin contraceptive pill, antiandrogens, or metformin to treat insulin resistance and prediabetes, GLP-1 analogs are also used widespread as anti-obesity drugs. Despite their high cost, they are demonstrating higher efficacy compared with metformin [140] administered alone or in combination with metformin [145]. Further studies are certainly needed to validate their efficacy in managing body weight but also on other metabolic and gynecological disorders typical of these patients. In recent years, attention is also being focused on other research areas such as genetics, epigenetics, the gut microbiota, and metabolomics related to PCOS.

Women with PCOS often show intestinal microbiota disorders, characterized by lower diversity and an altered intestinal and phylogenetic profile, and by an altered intestinal barrier, which leads to the activation of chronic inflammation and production of various molecular metabolites involved in the disease and clinical phenotype development of some patients with PCOS; this appears to be related to a diet high in sugar and fat found in women with PCOS [128, 146].

A systemic analysis found that the most common bacterial imbalances in PCOS patients involve *Bacteroidaceae*, *Coprococcus*, *Bacteroides*, *Prevotella*, *Lactobacillus*, *Parabacteroides*, *Escherichia*/*Shigella*, and *Faecalibacterium prausnitzii* [147].

Thus, nutritional intervention or the synergistic effect of diet and supplementation with probiotics, prebiotics, or synbiotics to promote bacterial diversity and enrichment of beneficial species such as *Bifidobacterium* and *Lactobacillus* could help improve the clinical picture in women with PCOS [147].

Further studies are certainly needed to validate the efficacy of pharmacological and nonpharmacological treatments, which are increasingly widespread.

## Conclusion

Lifestyle interventions have been shown to improve PCOS symptoms.

Therefore, a lifestyle change characterized by healthy eating and regular physical activity should be recommended for all women with PCOS to improve lifelong health and well-being by optimizing hormonal outcomes, overall health, and life quality. There is no single nutritional strategy for weight management; different nutritional approaches such as the Mediterranean or ketogenic diet, indeed, can induce the same improvement in nutritional status. Therefore, it is essential to evaluate the most suitable nutritional strategy based on the health state of each patient. However, although the benefits of KD have been, widely, reported, long-term compliance with KD is a limiting factor, making the diet unsustainable, also due to the important dietary restrictions necessary to induce ketosis. Furthermore, an emerging therapeutic strategy for PCOS includes also probiotics, prebiotics, and synbiotics administration, to improve the microbiome generally altered in PCOS women. However, to date, there are still poor data to support the efficacy, safety, and long-term health benefits of a specific nutritional approach, and the effects of long-term dietary management of PCOS need to be tested and verified through further studies. It seems clear, therefore, that to date, there is still no ideal or best treatment for PCOS. Identifying, for each patient, the prevalent factors and managing the aspects that most characterize the phenotype and cause discomfort to the woman with PCOS, through combined therapeutic strategies acting simultaneously on several aspects, appears to be the best way.
